# Supplementary material for: Comparative effectiveness and safety of insulin reference biologics versus biosimilars for types 1 and 2 diabetes mellitus: Protocol for a systematic review of real-world studies
Source: PLoS One. 2025 Jul 30;20(7):e0329299. doi: 10.1371/journal.pone.0329299 (PMC12310029; doi:10.1371/journal.pone.0329299)
Supplement: S1 Appendix — (DOCX) [file pone.0329299.s001.docx]

**S1 Appendix: Comparison with Past Reviews**

| **Systematic review** | **Population** | **Intervention/Exposure** | **Comparator** | **Outcomes** |
| --- | --- | --- | --- | --- |
| **Included RCTs and observational studies** | | | | |
| This planned review | Adults (aged ≥18 years) with T1DM or T2DM | Biosimilar insulins | Their respective reference products | Effectiveness, safety, immunogenicity |
| Tricco *et al.* (2021)(1) | Adults (aged ≥16 years) with T1DM | Ultra-long-/long-/intermediate-acting insulin as part of basal/bolus therapy (excluding pre-mixed insulin) | Ultra-long-/long-/intermediate-acting insulin, biosimilar insulin, no treatment | Efficacy, safety |
| Barbier *et al.* (2020)(2) | Patients who switch between a biosimilar and its reference product | Biosimilars approved in Europe, including biosimilar insulins | Their respective reference products | Efficacy, safety, immunogenicity |
| McKinnon et al. (2018)(3) | Patients who switch between a biosimilar and its reference product | Biosimilars, including biosimilar insulins | Their respective reference products | Efficacy, safety, immunogenicity |
| **Focused on RCTs (may have included preclinical and extension studies, but did not include observational studies)** | | | | |
| Herndon *et al.* (2023)(4) | Patients who switch between a biosimilar and its reference product | Biosimilars approved in the United States, including biosimilar insulins | Their respective reference products | Safety, immunogenicity |
| Yang *et al.* (2022)(5) | Patients with diabetes mellitus | Biosimilar insulins | Their respective reference products | Efficacy, safety, immunogenicity |
| Ampudia-Blasco (2020)(6) | Patients with diabetes | Biosimilar insulins, faster-acting insulin aspart, smart insulin | (For biosimilar insulins) their respective reference products, (for faster-acting insulin aspart and smart insulin) not specified | Pharmacokinetics, pharmacodynamics, efficacy, safety |
| Madenidou *et al.* (2018)(7) | Adults with T2DM | Basal insulin analogues (excluding insulin peglispro, NPH insulin, and pre-mixed insulin) | Basal insulin analogues | Efficacy, safety |
| Tieu *et al.* (2018)(8) | Adults | Biosimilar insulins | Their respective reference products | Pharmacokinetics, pharmacodynamics, efficacy, safety, immunogenicity |
| Yamada *et al.* (2018)(9) | Adults (aged ≥18 years) with T1DM or T2DM | Biosimilar insulins | Their respective reference products | Efficacy, safety, immunogenicity |

RCT: randomized controlled trial, T1DM: type 1 diabetes mellitus, T2DM: type 2 diabetes mellitus

**References**

1. Tricco AC, Ashoor HM, Antony J, Bouck Z, Rodrigues M, Pham B, et al. Comparative Efficacy and Safety of Ultra-Long-Acting, Long-Acting, Intermediate-Acting, and Biosimilar Insulins for Type 1 Diabetes Mellitus: a Systematic Review and Network Meta-Analysis. Journal of General Internal Medicine. 2021;36(8):2414-26.

2. Barbier L, Ebbers HC, Declerck P, Simoens S, Vulto AG, Huys I. The Efficacy, Safety, and Immunogenicity of Switching Between Reference Biopharmaceuticals and Biosimilars: A Systematic Review. Clinical Pharmacology & Therapeutics. 2020;108(4):734-55.

3. McKinnon RA, Cook M, Liauw W, Marabani M, Marschner IC, Packer NH, et al. Biosimilarity and Interchangeability: Principles and Evidence: A Systematic Review. BioDrugs. 2018;32(1):27-52.

4. Herndon TM, Ausin C, Brahme NN, Schrieber SJ, Luo M, Andrada FC, et al. Safety outcomes when switching between biosimilars and reference biologics: A systematic review and meta-analysis. PLOS ONE. 2023;18(10):e0292231.

5. Yang L-J, Wu T-W, Tang C-H, Peng T-R. Efficacy and immunogenicity of insulin biosimilar compared to their reference products: a systematic review and meta-analysis. BMC Endocrine Disorders. 2022;22(1).

6. Ampudia-Blasco FJ. Biosimilars and Novel Insulins. Am J Ther. 2020;27(1):e52-e61.

7. Madenidou A-V, Paschos P, Karagiannis T, Katsoula A, Athanasiadou E, Kitsios K, et al. Comparative Benefits and Harms of Basal Insulin Analogues for Type 2 Diabetes: A Systematic Review and Network Meta-analysis. Ann Intern Med. 2018;169(3):165-74.

8. Tieu C, Lucas EJ, Depaola M, Rosman L, Alexander GC. Efficacy and safety of biosimilar insulins compared to their reference products: A systematic review. PLOS ONE. 2018;13(4):e0195012.

9. Yamada T, Kamata R, Ishinohachi K, Shojima N, Ananiadou S, Nom H, et al. Biosimilar vs originator insulins: Systematic review and meta-analysis. Diabetes Obes Metab. 2018;20(7):1787-92.
