# Supplementary material for: Comparative effectiveness and safety of insulin reference biologics versus biosimilars for types 1 and 2 diabetes mellitus: Protocol for a systematic review of real-world studies
Source: PLoS One. 2025 Jul 30;20(7):e0329299. doi: 10.1371/journal.pone.0329299 (PMC12310029; doi:10.1371/journal.pone.0329299)
Supplement: S3 Appendix — (DOCX) [file pone.0329299.s003.docx]

**S3 Appendix: Draft Eligibility Form**

If you answer “No” to any of the following questions, exclude the search result. Otherwise, include the search result.

1. Does this study include **adult** patients (at least 18 years old) with **type 1 or 2 diabetes mellitus**?

Yes

No

Unclear

1. Is this an **observational** study (e.g., cohort, case-control, within-subject, cross-sectional, interrupted time series, controlled before and after) or **open-label pragmatic randomized controlled trial**? At the full-text screening step, use the PRECIS-2 tool to help determine if potentially relevant open-label randomized controlled trials are pragmatic.

Yes

No

Unclear

1. Are patients treated with **insulin** (aspart, degludec, detemir, glargine, glulisine, icodec, isophane, lente, lispro, NPH, semilente, ultralente)?

Yes

No

Unclear

1. Does the study compare insulin **biosimilars** with their **respective reference products**?

Yes

No

Unclear

1. Does the study report **at least one** of the following outcomes:

| **Effectiveness** |
| --- |
| - Change in glycated hemoglobin (HbA1c) |
| - Change in fasting plasma glucose (FPG) |
| - Time in range |
| - Microvascular complications   - Retinopathy   - Nephropathy   - Neuropathy |
| - Health-related quality of life |
| **Safety** |
| - Physician visits, emergency department visits, or hospital admissions for:   - Hypoglycemia   - Hyperglycemia   - Diabetic ketoacidosis |
| - Weight gain |
| - Immunogenicity |
| - Injection site reactions |
| - Incident cancers |

Yes

No

Unclear
