# Supplementary material for: Comparative effectiveness and safety of insulin reference biologics versus biosimilars for types 1 and 2 diabetes mellitus: Protocol for a systematic review of real-world studies
Source: PLoS One. 2025 Jul 30;20(7):e0329299. doi: 10.1371/journal.pone.0329299 (PMC12310029; doi:10.1371/journal.pone.0329299)
Supplement: S4 Appendix — (DOCX) [file pone.0329299.s004.docx]

**S4 Appendix: Targeted Website Search**

| **#** | **Organization** | **Link** |
| --- | --- | --- |
| **Regulatory Agencies** | | |
| 1 | Health Canada | https://search.open.canada.ca/opendata/?_ organization _limit=0&organization=hc-sc |
| 2 | US Food and Drug Administration (FDA) | https://www.fda.gov/ |
| 3 | European Medicines Agency (EMA) | https://www.ema.europa.eu/en |
| 4 | Medicines and Healthcare products Regulatory Agency (MHRA) | https://www.gov.uk/government/organisations/medicines-and-healthcare-products-regulatory-agency |
| 5 | Therapeutic Goods Administration (TGA) | https://www.tga.gov.au/ |
| 6 | Pharmaceuticals and Medical Devices Agency (PMDA) | https://www.pmda.go.jp/english/ |
| **Health Technology Assessment Agencies** | | |
| 7 | Canada’s Drug Agency (CDA) | https://www.cda-amc.ca/ |
| 8 | Institute for Clinical and Economic Review (ICER) | https://icer.org/ |
| 9 | National Institute for Health and Care Excellence (NICE) | https://www.nice.org.uk/ |
| 10 | Institute for Quality and Efficiency in Health Care (IQWiG) | https://www.iqwig.de/en/ |
| 11 | Haute Autorité de Santé (HAS) | https://www.has-sante.fr/jcms/pprd_2986129/en/home |
| 12 | Prescrire International | https://english.prescrire.org/en/Summary.aspx |
