# Supplementary material for: Comparative effectiveness and safety of insulin reference biologics versus biosimilars for types 1 and 2 diabetes mellitus: Protocol for a systematic review of real-world studies
Source: PLoS One. 2025 Jul 30;20(7):e0329299. doi: 10.1371/journal.pone.0329299 (PMC12310029; doi:10.1371/journal.pone.0329299)
Supplement: S5 Appendix — (DOCX) [file pone.0329299.s005.docx]

**S5 Appendix: MEDLINE Search Strategy**

MEDLINE (Ovid)

Search conducted on November 29, 2024

| **#** | **Query** | **Records retrieved** |
| --- | --- | --- |
| 1 | exp Diabetes Mellitus/ | 538090 |
| 2 | diabet*.tw,kf. | 848013 |
| 3 | insulin defic*.tw,kf. | 3566 |
| 4 | DM.tw,kf. | 66761 |
| 5 | T1DM.tw,kf. | 7383 |
| 6 | IDDM.tw,kf. | 6934 |
| 7 | T2DM.tw,kf. | 38621 |
| 8 | NIDDM.tw,kf. | 6999 |
| 9 | exp Hyperglycemia/ | 42825 |
| 10 | hyperglyc?emi*.tw,kf. | 81293 |
| 11 | impaired glucose tolerance.tw,kf. | 12709 |
| 12 | or/1-11 | 974472 |
| 13 | insulins/ or biphasic insulins/ or exp insulin, longacting/ or exp insulin, short-acting/ or exp insulin/ | 208286 |
| 14 | (insulin* or actrapid or afrezza or biosulin or entuzity or humulin* or hypurin or iletin or monotard or myxredlin or novolin* or rapitard or velosulin).tw,kf. | 436358 |
| 15 | (aspart* or fiasp or GP40071 or GP40081 or kirsty or kixelle or MYL-1601D or novolog or novomix or novorapid or SAR341402 or trurapi or truvelog).tw,kf. | 127914 |
| 16 | (degludec* or NN1250 or ryzodeg or tresiba or xultophy).tw,kf. | 938 |
| 17 | (detemir* or levemir or NN-304 or NN304).tw,kf. | 1049 |
| 18 | (efsitora* or LY3209590).tw,kf. | 15 |
| 19 | (glargine* or abasaglar or abasria or basaglar or basalin or basalog or FFP-112 or GP40061 or HOE 901 or HOE901 or lantus or LY2963016 or MK-1293 or MYL-1501D or optisulin or rezvoglar or semglee or soliqua or toujeo).tw,kf. | 3389 |
| 20 | (glulisine* or apidra or HMR1423).tw,kf. | 354 |
| 21 | (icodec* or awiqli or NN1436).tw,kf. | 87 |
| 22 | (isophane* or insulatard or neutral protamine hagedorn or nph* or protaphane).tw,kf. | 6492 |
| 23 | (lente* or semilente* or ultralente*).tw,kf. | 1022 |
| 24 | (lispro* or admelog or GP40021 or humalog or liprelog or liprolog or lyumjev or SAR342434).tw,kf. | 1347 |
| 25 | (tregopil* or IN-105).tw,kf. | 5852 |
| 26 | (doublestar or flextouch or flexpen or innolet or kwikpen or solostar).tw,kf. | 127 |
| 27 | or/13-26 | 605221 |
| 28 | Biosimilar Pharmaceuticals/ | 3823 |
| 29 | biosimilar*.tw,kf. | 5987 |
| 30 | bioequivalent.tw,kf. | 2993 |
| 31 | subsequent entry.tw,kf. | 231 |
| 32 | follow-on.tw,kf. | 1702 |
| 33 | biocomparable*.tw,kf. | 6 |
| 34 | similar biologic*.tw,kf. | 2139 |
| 35 | (originator* or innovator* or non-innovator* or reference product* or reference biologic*).tw,kf. | 6948 |
| 36 | or/28-35 | 17392 |
| 37 | 12 and 36 | 506 |
| 38 | 27 and 36 | 474 |
| 39 | 37 or 38 | 725 |
