# Supplementary material for: Comparative effectiveness and safety of insulin reference biologics versus biosimilars for types 1 and 2 diabetes mellitus: Protocol for a systematic review of real-world studies
Source: PLoS One. 2025 Jul 30;20(7):e0329299. doi: 10.1371/journal.pone.0329299 (PMC12310029; doi:10.1371/journal.pone.0329299)
Supplement: S6 Appendix — (DOCX) [file pone.0329299.s006.docx]

**S6 Appendix: Draft Data Collection Form**

| **General Article Information** | |
| --- | --- |
| Title |  |
| First author |  |
| Journal |  |
| Year |  |
| DOI |  |
| Country(s) |  |
| Funding source(s) |  |
| **Study Overview** | |
| Study design |  |
| Study period |  |
| Length of follow-up |  |
| Primary study aim/objective |  |
| **Data Sources** | |
| Setting (e.g., multinational, national, state/province, institution) |  |
| Data sources (e.g., administrative claims, electronic health records, registries) |  |
| Data type (e.g., prescribing, dispensing, purchasing) |  |
| **Methodology** | |
| Data analysis |  |
| Statistical models |  |
| Covariates measured |  |
| **Results** | |
| **Patient characteristics** | |
| Number of patients |  |
| Age (mean and standard deviation) |  |
| Sex (%) |  |
| Type of diabetes (%) |  |
| Duration of diabetes (mean and standard deviation) |  |
| Baseline glycosylated hemoglobin (HbA1c) (mean and standard deviation) |  |
| **Drug Characteristics** | |
| International nonproprietary name(s) |  |
| Biosimilar(s) |  |
| Reference product(s) |  |
| Delivery (e.g., injection, pump) |  |
| Dosage(s) |  |
| **Outcomes** | |
| Effectiveness:   - Change in glycosylated hemoglobin (HbA1c) - Change in fasting plasma glucose (FPG) - Time in range - Microvascular complications (retinopathy, nephropathy, neuropathy) - Health-related quality of life |  |
| Safety:   - Physician visits, emergency department visits, or hospital admissions for hypoglycemia, hyperglycemia, or diabetic ketoacidosis - Weight gain - Immunogenicity - Injection site reactions - Incident cancers |  |
| Key findings |  |
| Conclusions |  |
