# Supplementary material for: Comparative effectiveness and safety of insulin reference biologics versus biosimilars for types 1 and 2 diabetes mellitus: Protocol for a systematic review of real-world studies
Source: PLoS One. 2025 Jul 30;20(7):e0329299. doi: 10.1371/journal.pone.0329299 (PMC12310029; doi:10.1371/journal.pone.0329299)
Supplement: S7 Appendix — (DOCX) [file pone.0329299.s007.docx]

**S7 Appendix: Draft Summary Table of Included Studies**

| **Authors** | **Insulin product** | **Funding source** | **Diabetes mellitus type** | **Number of patients** | **Follow-up duration** | **Relevant outcomes** | **Authors’ conclusions** |
| --- | --- | --- | --- | --- | --- | --- | --- |
| Open-label pragmatic randomized controlled trials | | | | | | | |
|  |  |  |  |  |  |  |  |
|  |  |  |  |  |  |  |  |
| Cohort studies | | | | | | | |
|  |  |  |  |  |  |  |  |
|  |  |  |  |  |  |  |  |
| Case-control studies | | | | | | | |
|  |  |  |  |  |  |  |  |
|  |  |  |  |  |  |  |  |
