# Supplementary material for: Comparative effectiveness and safety of insulin reference biologics versus biosimilars for types 1 and 2 diabetes mellitus: Protocol for a systematic review of real-world studies
Source: PLoS One. 2025 Jul 30;20(7):e0329299. doi: 10.1371/journal.pone.0329299 (PMC12310029; doi:10.1371/journal.pone.0329299)
Supplement: Attachment — Submitted filename: Response to Editor_Biosimilar Insulins.docx [file pone.0329299.s009.docx]

May 29, 2025

Dear Dr. Dharmagadda:

Thank you for taking the time to review our systematic review protocol. We genuinely appreciate your thoughtful feedback and have made the suggested changes. A point-by-point response to the comments is provided below.

**1. Clarify the Scope of “Real-World Studies”: Provide explicit thresholds or examples based on the PRECIS-2 domains to support consistent judgement**

- The PRECIS-2 tool was originally designed to help researchers prospectively design their randomized controlled trials (RCTs). However, the PRECIS-2 authors have recognized that it can also be used to retrospectively assess pragmatism in RCTs for literature reviews.
- As we mention on lines 113-114, there is no simple dichotomous threshold for distinguishing pragmatic versus explanatory RCTs. It is not recommended to sum up the domain scores, rank-order the domains by importance, or set a minimum number of domains to determine pragmatism.
- Consistent with these recommendations, we will not apply an explicit threshold. The decision on whether an RCT is pragmatic will be guided by PRECIS-2 scores but ultimately up to the reviewers.
- Nevertheless, we agree that we can be more explicit to guide reviewers on how to use PRECIS-2 to inform their judgement and discussion. Furthermore, to promote transparency and reproducibility, we will publish our PRECIS-2 scores for candidate RCTs.

Changes:

- Added to the Methods: “As our objective is to evaluate insulin use in the real world, we will exclude experimental studies that are not open-label pragmatic RCTs. The PRECIS-2 tool helps distinguish between pragmatic versus explanatory RCTs.(35) However, even when using this tool, there is no simple dichotomous threshold for making this distinction; reviewers will use a combination of PRECIS-2 and their judgement. *We appreciate that the PRECIS-2 authors do not recommend dichotomizing RCTs as a whole as pragmatic or explanatory.(39) Our use of PRECIS-2 to exclude explanatory RCTs is to avoid research waste through duplication of effort, as there have been systematic reviews of RCTs on similar topics as recent as 2023,(19) while fulfilling our objective of summarizing the available real-world evidence.*”
- Added to the Methods: “For open-label pragmatic RCTs, reviewers will use the PRECIS-2 tool to help decide whether they are pragmatic.(38, 39) *Trials that score 4 or 5 out of 5 across many domains are more likely to be pragmatic RCTs. Domains with high scoring variation by reviewers (e.g., by 2 levels or more) will be resolved by discussion or a third reviewer.* Disagreements *on the overall pragmatism of studies* will *also* be resolved by discussion or a third reviewer. *The PRECIS-2 scores for candidate RCTs, including those that were ultimately excluded, will be published with the completed systematic review.*”

**2. Update and Expand Literature Coverage: While you mentioned that past systematic reviews are outdated (last up to 2019), it may be helpful to quantify the preliminary yield of the updated literature search and mention any especially recent high-impact real-world studies, if already identified. Consider including a table comparing key attributes of past reviews versus this planned review.**

- Thank you for this feedback. We agree that these changes can help highlight the novelty.
- Quantifying the preliminary yield of our literature search, especially by mentioning recent and high-impact studies, will demonstrate the need for our review. We now cite several relevant studies that we came across in our feasibility search in the Introduction. However, we are cautious with the wording, as our feasibility search was only a scan of published literature and may underrepresent the growing body of evidence since 2019.
- Furthermore, we have added a new table to the appendix (S2 Appendix) that compares the past reviews with this planned review.

Changes:

- Added to the Introduction: “Existing biosimilar insulin reviews(25-27) that include real-world studies have only found three observational studies on this topic.(28-30) However, these systematic reviews did not cover both types of diabetes or all currently available biosimilar insulins. *We have summarized the differences between these past reviews and our planned review in S2 Appendix.*”
- Added to the Introduction: “We anticipate additional real-world studies in the past six years, *such as those by Pitlick et al. (2020), Pham et al. (2022), and AlRuthia et al. (2022).(34-36)*”
- Added S2 Appendix: Comparison with past reviews

**3. Risk of Bias Assessment and Heterogeneity: Given the inclusion of both observational and pragmatic RCTs, the heterogeneity could be substantial. Even if meta-analysis is not planned, outline how heterogeneity will be qualitatively assessed (e.g., using a matrix of study designs/outcomes).**

- Thank you for this suggestion. To qualitatively assess heterogeneity, we will present our findings in a summary table with the following headers, sorted by study design:
  - Author (year)
  - Insulin product
  - Funding source
  - Diabetes mellitus type
  - Number of patients
  - Follow-up duration
  - Relevant outcomes
  - Authors’ conclusions
- This summary table can illustrate whether different study designs consistently report different patient characteristics and outcomes. We have added this shell table to the appendix (S7 Appendix).

Changes:

- Added to the Methods: “Therefore, we anticipate a high level of heterogeneity in study design (comparators, outcomes, user definitions, bias, etc.) that would preclude a valid pooling of results. *To qualitatively assess this heterogeneity, we will present the above findings in a summary table, organized by study design. A draft summary table is presented in S7 Appendix.*”
- Added S7 Appendix: Draft summary table of included studies

**4. Language: A few minor typographical and formatting issues could be improved.**

- We identified some formatting issues and made the edits listed below.
- Of note, “aspart” (drug name), “OAIster” (catalog name), “citationchaser” (R package), and “textword” (field-specific terminology) have been marked by Microsoft Word as spelling mistakes. We confirm that these are the correct spellings.

Changes:

- Changed reference list font to Times New Roman, the font size to 12, and aligned left.
- Changed all rows in the tables in S3 and S6 Appendices to be single-spaced.
- Changed all columns in the table in S6 Appendix to be left-aligned.

Thank you for considering our manuscript.

Warm regards,

Martin Ho, PharmD

University of Toronto
